# Effects of Three Preseason Training Programs on Speed, Change-of-Direction, and Endurance in Recreationally Trained Soccer Players

**DOI:** 10.3389/fphys.2021.719580

**Published:** 2021-09-17

**Authors:** Jérôme Koral, Jaume Lloria Varella, Fernando Lazaro Romero, Clément Foschia

**Affiliations:** ^1^Laboratoire Interuniversitaire de Biologie de la Motricité, Université Jean Monnet, Saint-Etienne, France; ^2^Albacete Balompie SAD, Albacete, Spain; ^3^Department of Clinical and Exercise Physiology, Sports Medicine Unit, Faculty of Medicine, University Hospital of Saint-Etienne, Saint-Etienne, France

**Keywords:** repeated sprints, change-of-direction, power, strength, endurance, soccer

## Abstract

**Background:** Modern coaches experience a drastic reduction of the available training time with an increasingly large number of competitions during the competitive season. Thus, they must choose wisely the most efficient methods to improve the physical fitness of their players during the preseason. Among all the methods, this study compared the effects of plyometric training (PT), sprint interval training (SIT), and small-sided games (SSGs) on the performance of recreationally trained soccer players.

**Methods:** Seventy-three participants were randomly assigned in one of the three experimental groups (i.e., PT [*n* = 23], SIT [*n* = 26] or SSGs [*n* = 24]) and completed two sessions per week for a total of 3 weeks. Meanwhile, the whole group maintained their habitual soccer-specific training program who do not interfere in the preparation of the season. Repeated sprint ability (RSA), maximal aerobic speed (MAS), and a 30-m sprint were assessed at baseline (PRE) and post-training (POST).

**Results:** Performance in SSGs decreased for the average speed from 0 to 10 m (*V*_0−10m_; −0.84 km h^−1^, −4 ± 5%, *p* < 0.001), the maximal distance (*D*_max_) covered in the 30-s RSA test (−3.65 m, −3 ± 6%, *p* < 0.01) and MAS (−0.52 km h^−1^, −3 ± 6%, *p* < 0.01). PT increased the mean distance (*D*_mean_) covered in the 30-s RSA test (+5.98 m, 5 ± 4%, *p* < 0.001) and MAS (+0.58 km h^−1^, 7 ± 5%, *p* < 0.01) while an improvement of all parameters but the maximal sprint speed reached during the 30-m trip (*V*_max_) was found in the SIT group (*V*_0−10m_: +1.462 km h^−1^, 8 ± 5%, *p* < 0.001; *D*_max_: +7.89 m, 6 ± 5%, *p* < 0.001; *D*_mean_: +8.69 m, 7 ± 5%, *p* < 0.001 and MAS: +1.74 km h^−1^, 12 ± 8%, *p* < 0.001). All SSG POST values were significantly lower than PT and SIT (*p* < 0.01). *D*_mean_ and MAS in POST were also significantly higher in SIT than in the PT group (*p* < 0.001).

**Conclusion:** This study suggests that both PT and SIT could be a better alternative to SSGs to boost performances during preseason. Moreover, SIT seems to produce higher improvements in physical performances than PT.

## Introduction

Soccer is an invasive team field game with an intermittent activity profile (Drust et al., 2000), which is characterized by around 1,200 acyclical, very variable, and unpredictable actions (Iaia et al., 2009). Those bouts involve various types of linear sprints interspersed with rapid changes-of-direction (CoD), decelerations, sudden starts, stops, jumps, kicks, and tackles (Bloomfield et al., 2007; Iaia et al., 2009; Pavillon et al., 2021). More specifically, it is well-established that during a professional soccer match, an average of 80% of physical activities is considered as low-to-moderate intensities such as standing, walking, or jogging, and the remaining 20% of physical activities are classified as high-intensity activities (running 12–20 km h^−−1^) or sprints (Mohr et al., 2003; Bloomfield et al., 2007; Pavillon et al., 2021). Despite those last intense actions that have been reported to occur only each 60 s for high intensities (Strudwick et al., 2002) or every 4–5 min for the sprints (Drust et al., 2000; Strudwick et al., 2002; Rampinini et al., 2007a), they are crucial and nearly always precede match-winning actions such as goal situation (Faude et al., 2012).

Consequently, both coaches and researchers have focused on determining the optimal training methods (i.e., strength training and repeated sprint training) for the development of CoD and repeated maximal linear sprint performance, which constitute the key physical qualities in soccer (Cometti et al., 2001; Markovic et al., 2007; Faude et al., 2012; Trecroci et al., 2016, 2020; Pavillon et al., 2021). To do so, three main approaches are usually considered. The first two are inspired by track and field training methods, one focused on strength training and another on traditional running-based conditional exercises, whereas the last one is more ecological and tries to mimic as much as possible the game conditions.

The strength training approach is based on several studies that reported strong correlations between lower-body strength and short-sprint performance (Seitz et al., 2014). For instance, maximal strength is reported to be one of the most important factors in maximizing power output (Cronin and Hansen, 2005; Weyand et al., 2006, 2010; Thapa et al., 2021). Hence, there is no doubt about the beneficial effects of strength training to increase short-sprint performance (Styles et al., 2016) and/or sprint ability (Markovic et al., 2007). Up to now, practitioners and researchers have mostly focused on heavy resistance training and plyometric training (PT) in soccer (De Hoyo et al., 2016; Bauer et al., 2019; Ramirez-Campillo et al., 2020). Most of them tend to agree that PT, due to a rapid eccentric to concentric muscle contraction, represents a method of choice when aiming to enhance a wide range of athletic performance particularly those involving the stretch-shortening cycle (SSC) such as jumping, sprinting, and agility (Fatouros et al., 2000; Markovic et al., 2007; Slimani et al., 2016; Negra et al., 2020). Thapa et al. (2021) even considered PT as a bridge between strength and speed. Nevertheless, as the distance traveled during a game is comprised between 9,995 and 11,233 m (Rampinini et al., 2007a), soccer relies primarily on aerobic metabolism for energy (Strøyer et al., 2004). In addition, when measuring the effects of PT on endurance in soccer, the results are more contrasted.

The second approach is a direct consequence of the aforementioned necessity of developing endurance. Coaches usually dedicate a non-negligible amount of time during the preseason to improve this capacity because they are aware that the aerobic pathway and more specifically VO_2_max are of crucial importance in soccer (Mallo and Navarro, 2008). For instance, Helgerud et al. (2001) reported that the higher the VO_2_max and running economy, the better the performances during the game. Moreover, the optimization of VO_2_max has been reported (i) to allow better repeat sprints (Glaister, 2005), (ii) to better recover between each sprint (Aziz et al., 2007; Brown et al., 2007), and therefore (iii) to maintain higher sprint velocity during the game (Bishop and Edge, 2006). In addition, traditional running-based training programs have been broadly studied, especially when based on very high intensities. Those methods seem to be focused on enhancing maximal performance while reducing the total workout time. Among these techniques, sprint interval training (SIT) seems particularly interesting, because (i) it is nearly costless as no special equipment is needed; (ii) it generates improvements not only in all muscle energy pathways (i.e., aerobic and anaerobic; Parra et al., 2000; Rodas et al., 2000; Ross and Leveritt, 2001; Burgomaster et al., 2007; Weston et al., 2014; Milanović et al., 2015) but also on endurance, strength, power, and speed performance (Taylor et al., 2015; Koral et al., 2018). Nevertheless, sports science literature has quite neglected the possible use of this potentially useful explosive exercise for training purposes when various studies suggest that sprint training could lead to improvements in human muscle power capabilities (Malisoux et al., 2005) and dynamic athletic performance in both concentric and SSC muscle function (Markovic et al., 2007). Additionally, repeated sprints have been reported to be more efficient at improving short-sprint performance than methods such as PT (Taylor et al., 2015). Yet, soccer coaches still do not often use the SIT or strength training as they may find it quite distant from their specific activity and/or too demanding in preseason.

In fact, due to the professionalization of sport that has led to a sharp increase in the number of competitions while reducing training and recovery times (Issurin, 2008, 2010), coaches progressively left out those traditional running-based conditional exercises inspired by track and field training methods (Moran et al., 2019) to focus on more ecological methods but equally effective on fitness and football-specific performance (Hill-Haas et al., 2011; Bujalance-Moreno and Garc-a-Pinillos, 2017) such as small-sided games (SSGs). Those SSGs reproduce on a smaller scale of all the aspects of soccer that are required during competitions (Clemente and Sarmento, 2020; Clemente et al., 2020, Younesi et al., 2021). They encompass the psychological, physical, technical, and tactical aspects of the game (Clemente et al., 2012) and thus are much more accepted by soccer players (Jastrzebski et al., 2014). Moreover, recent studies reported strong relationships between traditional aerobic fitness tests with external and internal training load measures during SSGs (Stevens et al., 2016; Owen et al., 2020). Nevertheless, other authors suggested that SSGs themselves may not be enough to promote the same patterns of the required physical demands during a soccer match, mainly due to the reduced frequency of high-intensity distance-based metrics of this training approach (Lacome et al., 2017; Younesi et al., 2021). For instance, Hoff and Helgerud (2004) showed that the most skilled the players are, the lower the aerobic fitness adaptations will be elicited by SSGs. Conversely, SSGs can also be counterproductive for less-skilled players as they may not be able to consistently sustain the technical skill or tactical proficiency to achieve and maintain the required metabolic strain (Castagna et al., 2004). Moreover, Casamichana et al. (2012) reported a low number of sprints performed during SSGs combined with high volumes of aerobic stimuli. This can result in an acute reduction in sprint velocity (Katis and Kellis, 2009) or no improvements in sprint ability (Hill-Haas et al., 2009) after the use of SSGs as the conditioning strategy.

Rampinini et al. (2007a, 2011) characterized soccer as an intermittent activity where brief bouts of high-intensity running are interspersed with longer periods of low-intensity exercise. In other words, when pursuing performance in soccer, speed, power, and aerobic fitness are intimately linked and need to be all upgraded. Therefore, the aim of this study was to compare the effects on physical performance of the three different approaches (i.e., PT, SIT, and SSGs) performed in the field in recreationally trained soccer players. We hypothesized that soccer players would differently beneficiate from the different types of training, being SIT the most impactful in the measured variables.

## Methods

### Athletes

Seventy-three recreationally trained soccer players (3–4 soccer training sessions per week) volunteered to take part in the experiment. Participants were randomly assigned to one of the three training groups, namely, PT, SIT, and SSGs. Mean (±SD) age, height, and maximal aerobic speed (MAS) are presented in Table 1. As those interventions were planned during the preseason, none of the players performed intense training (intermittent or not) during the month preceding this intervention. All training was included in addition to their usual soccer training program. The Universities Ethics Board and Human Research Ethics Committee approved this study (IRBN1042020/CHUSTE), and following a routine medical screening, participants were informed of the procedures to be employed and the associated risks and benefits of the intervention. An institutionally approved written consent form was provided and signed by all participants before any training or testing.

**Table 1:** Characteristics of the participants.

**Group**	**Participants (n)**	**Age**	**Mass**	**Height**	**Maximal aerobic**
		**(years)**	**(kg)**	**(m)**	**speed (km.h^**−1**^)**
SSGs	24	19.3 ± 5.1	67.9 ± 8.6	1.77 ± 0.07	14.3 ± 1.1
PT	23	19.5 ± 4.0	68.1 ± 7.5	1.77 ± 0.06	14.7 ± 1.2
SIT	26	19.2 ± 3.7	67.7 ± 7.1	1.76 ± 0.06	14.4 ± 1.0

*SSGs, small-sided games; PT, plyometric training; SIT, sprint interval training*.

#### Experimental Protocol

The experimental design included a familiarization procedure, pretests, 3 weeks of training (PT, SIT, or SSGs), and post-tests. Before each test and training session, participants completed a standardized warm-up consisting of light to medium muscular contractions (3 sets of 10 repetitions on knee extensors and knee flexors) and three sets of 25-m progressive runs (60, 70, and 80% of the maximal sprint speed). All familiarization, testing, and training sessions were conducted in the afternoon (5–7 P.M.) to avoid performance fluctuations due to circadian rhythms. Participants were encouraged to drink water before, during, and after each testing and training session. All participants were instructed not to deviate from their current, dietary habits, or hydration patterns throughout the duration of this study. They were asked to refrain from any other kind of exercise during the experiment.

#### Familiarization Procedures

Before taking part in any experimental trial (baseline measurements), all subjects performed familiarization trials to become oriented with all testing procedures. The familiarization also consisted of two to three maximal bouts of 30-s shuttle runs with 4 min of recovery between bouts.

#### Pre- and Post-Testing

**Figure 1:**
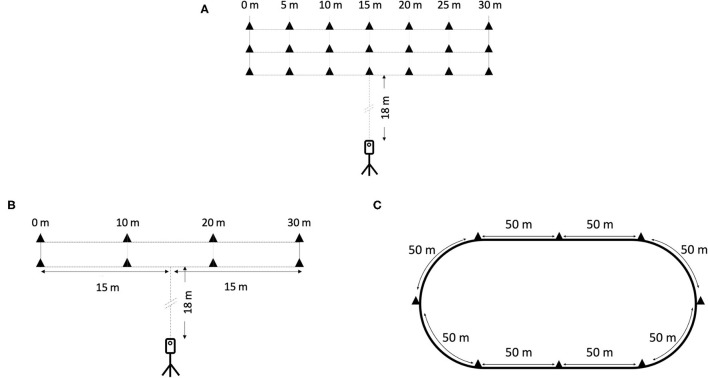
Representation of testing protocol (PRE and POST). **(A)** Repeated sprint ability test, **(B)** maximal sprint speed test, and **(C)** maximal aerobic speed test.

Baseline measurements for all subjects consisted of a repeated sprint ability (RSA) test (Figure 1A), and 48 h after, a maximal sprint speed test over 30 m (Figure 1B), and a MAS test (Figure 1C) interspersed by 20-min recovery. An experienced strength and conditioning coach supervised each test session and provided participants with strong verbal encouragement.

##### RSA Test

On a soccer pitch, each line was materialized by placing markers 5 m from each other for a total of 30 m (Figure 1A). Based on Koral et al. (2018), participants were asked to perform four bouts of 30 s all-out shuttle sprints during which they had to travel the greatest distance possible making trips of 5, 10, 15 m, and so on (Figure 1A). A 4-min recovery was fixed between each bout. All sprints were recorded with an iPhone 7 plus (Apple, 2017) mounted to a tripod at 240 Hz. Based on Romero-Franco et al. (2017), the iPhone was placed in a fixed position at 18 m from the pitch in the frontal plane and at the 15 m marker. The following two variables were obtained for each test session:

1. Maximal distance (*D*_max_): the longest total distance covered in a 30-s period.
2. Mean distance (*D*_mean_): the total distance of the session divided by the number of repetitions: *D*_mean_ = (*D*_max1_ + *D*_max2_ + *D*_max3_ + *D*_max4_)/4.

##### Maximal Sprint Speed Test

Participants were supervised and instructed to run as fast as possible over 30 m on a flat soccer pitch. They performed two 30-m sprints with 5-min rest. Markers were placed every 10 m (from 0 to 30 m). All sprints were recorded with an iPhone 7 plus (Apple, 2017) mounted to a tripod at 240 Hz. The iPhone was placed in a fixed position at 18 m from the track in the frontal plane and at 15 m from both starting and finishing lines (Figure 1B). Based on Romero-Franco et al. (2017), video parallax was corrected to ensure 10, 20, and 30 m split times were measured properly. Data were analyzed using the application *MySprint*, in which validity and reliability have been demonstrated (Romero-Franco et al., 2017) to determine the average speed from 0 to 10 m (*V*_0−10m_), and the maximal sprint speed reached during the 30-m trip (*V*_max_) which was determined as the fastest 10 m interval.

##### MAS Test

A continuous running multistage field test, known as the “University of Montreal Track Test” (Léger and Boucher, 1980), was utilized. This protocol was run on a 400-m flat running track, with markers located every 50 m along the track (Figure 1C). No warm-up was performed before the test, and the speed of the initial stage was set at 8 km^.^h^−1^ and increased by 1 km^.^h^−1^ every 2 min. The speed changes were indicated by audio cues from a prerecorded audio file. The test ceased when the subject fell 5 m short of the designated markers, or when the subject reached volitional failure. The validity and reliability of this test are well-established (Léger and Boucher, 1980).

#### Training Period

The training period started 2 days after baseline testing. Two weekly sessions were performed by each group (i.e., SSGs, PT, and SIT) within their usual soccer training on Mondays and Wednesdays, whereas Fridays were only dedicated to soccer. The total time of the training session (training condition + soccer or only soccer) varied from 90 to 100 min.

##### SSGs

Participants did a standard preseason based on SSGs on the field. Those SSGs were programmed with smaller fields and less players than during a traditional soccer game. Based on Rampinini et al. (2007b), SSGs were played with no goalkeepers, small goals, and free touches. In order to increase the training load in SSGs, the pitch area moved from large (1,728 m^2^) to small (480 m^2^), and the number of players was also reduced each week altering, therefore, the difficulty of the technical-tactical task. The training protocol of week 1 consisted of four sets of 4 min with 3 min of passive recovery between sets. In week 2, the number of sets was increased from 4 to 5. And in week 3, the time of work was extended from 4 to 5 min. The organization, modulations, and contents of the 3-week training period are presented in Table 2.

**Table 2:** Small-sided game-specific soccer training program distribution over the 3-week training.

**Week**	**Session**	**Game**	**Training**	**Inter sets**	**Total**	**Pitch**
		**design**	**prescription**	**passive rest**	**session time**	**dimensions (m)**
1	1	6 vs. 6	4 × 4 min	3 min	28 min	36 x 48
	2	6 vs. 6	4 × 4 min	3 min	28 min	36 x 48
2	3	4 vs. 4	5 × 4 min	3 min	32 min	30 x 34
	4	4 vs. 4	5 × 4 min	3 min	32 min	30 x 34
3	5	3 vs. 3	5 × 5 min	3 min	37 min	24 x 20
	6	3 vs. 3	5 × 5 min	3 min	37 min	24 x 20

##### PT

Participants had to perform five different kinds of exercises over the 3-week PT, namely, vertical jumps, squat jumps, countermovement jumps, horizontal squats on a sled (with or without the help of elastic bands), and drop jumps (Table 3). During the first three sessions, participants did three sets of five repetitions by exercise and went up to four sets of five repetitions in the last three sessions. Due to the high impacts generated during the drop jumps, the total load was lowered to three sets of three repetitions in sessions 1–3 and four sets of three repetitions in sessions 4–6 compared with the other exercises (Table 3). Vertical jumps and squat jumps were used to enhance the concentric muscle function when countermovement jumps and drop jumps were introduced to improve the SSC muscle function. The inclusion of a Swiss-ball in squat jumps and a sledge (assisted or not with an elastic band) in the horizontal squat during sessions 3–6 were proposed to exacerbate the velocity of each repetition by reducing the body weight (Samozino et al., 2018).

**Table 3:** Plyometric training program distribution over the 3-week training.

**Week**	**Session**		**Exercises**				**Rest between** **sets & exercises (min)**	**Total** **session** **time (min)**
		**Vertical** **jumps**	**Squat** **jumps**	**Counter** **movement jumps**	**Horizontal squats** **on sled**	**Drop** **jumps**		
1	1	3 x 5 on 40 cm box	3 x 5 at BW	3 x 5 at BW	-	3 x 3 from 20 cm at BW	2	~30
	2	3 x 5 on 40 cm box	3 x 5 with SB	-	3 x 5	3 x 3 from 20 cm at BW	2	~30
2	3	3 x 5 on 40 cm box	3 x 5 with SB	-	3 x 5 assisted with EB	3 x 3 from 20 cm at BW	2	~30
	4	4 x 5 on 40 cm box	4 x 5 at BW	4 x 5 at BW	-	4 x 3 from 20 cm at BW	2	~35
3	5	4 x 5 on 40 cm box	4 x 5 with SB	-	4 x 5	4 x 3 from 20 cm at BW	2	~35
	6	4 x 5 on 40 cm box	4 x 5 with SB	-	4 x 5 assisted with EB	4 x 3 from 20 cm at BW	2	~35

*BW, bodyweight; EB, elastic band; SB, Swiss ball*.

##### SIT

Following the distribution of the RSA test, the soccer pitch was divided by placing markers every 5 m and up to 30 m (Figure 1A). Each training session consisted of repeated 30 s of “all-out” shuttle run efforts interspersed by a period of 4-min of rest (Table 4). Regarding the RSA test, the instructions were to travel the greatest distance possible in 30 s making trips of 5, 10, 15 m, etc. SIT volume progressively increased from 4 to 7 bouts over the first five sessions and was reduced to four bouts in the last session (Table 4). Participants received strong verbal encouragement to continue running maximally without pacing throughout the 30 s bouts. Up to six players performed SIT simultaneously and, during the 4-min recovery period, they walked back to the start line where they waited for the following repetitions.

**Table 4:** Sprint interval training distribution over 3 weeks.

**Week**	**Session**	**Number of**	**Training sprint**	**Total session**
		**sprints**	**time (min)**	**time (min)**
1	1	4	2	14
	2	5	2.5	18.5
2	3	6	3	23
	4	6	3	23
3	5	7	3.5	27.5
	6	4	2	14
	Total	32	16	110

### Statistical Analysis

Data are presented as mean values ± SD in the text, tables, and figures and were analyzed using the STATISTICA 2007 version 8.0. (StatSoft, Inc., Tulsa, OK, USA) software. The normality assumption was verified using Shapiro-Wilk's test. In this study, the performance-related variables studied were analyzed using a two-factor mixed model design ANOVA to test the effect of the training along the time and the effect of each type of intervention on the participants (time × condition). Neuman-Keuls *post-hoc* was performed to determine between means differences if the ANOVA revealed a significant main effect or an interaction. The significance level was set at *p* < 0.05. Finally, the effect size was calculated using the partial eta square (η^2^p). Criteria for evaluating the effect size were 0.01 = small, 0.06 = medium, and 0.14 = large (Cohen, 1988).

## Results

**Figure 2:**
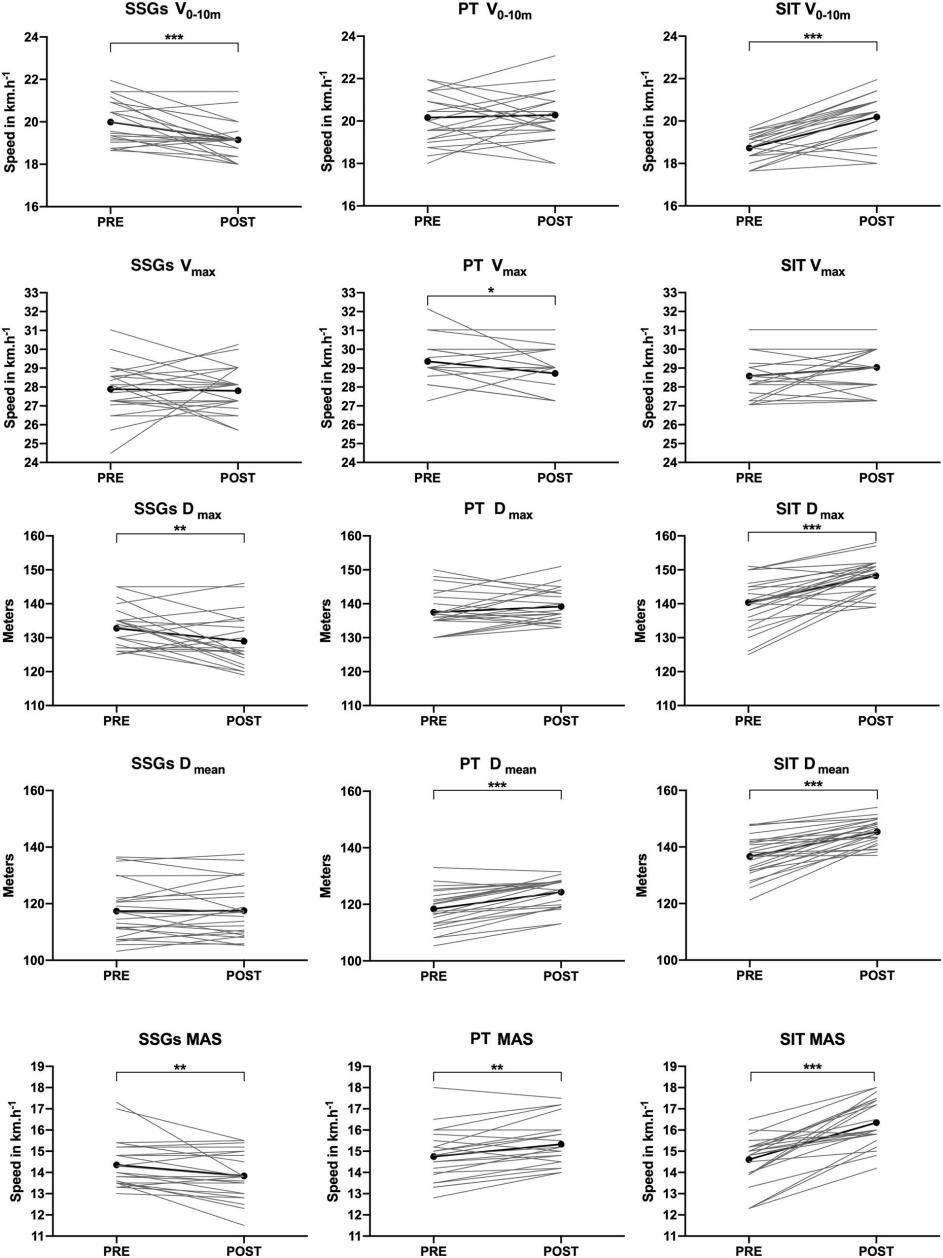
Individuals and mean PRE-POST variations in all parameters. SSGs, small-sided games; PT, plyometric training; SIT, sprint interval training. *V*_0−10m_: maximal speed from 0 to 10 m; *V*_max_: maximal sprint speed reached during the 30-m trip; *P*_max_: peak power output; *P*_mean_: mean power output; MAS: maximal aerobic speed. ^*^p < 0.05; ^**^p < 0.01; ^***^p < 0.001.

All individual mean PRE-POST changes are reported in Figure 2. All individual raw data, descriptive statistics, and effects are presented in Table 5.

**Table 5:** Raw data, descriptive statistics, and effects by groups.

**Descriptive statistics**	**Pre ± SD**	**Post ± SD**	**Mean difference**			
**V**_**0-10**_ **(km.h** ^**-1**^ **)**						
SIT	18.728 ± 0.631	20.190 ± 1.047	1.462			
PT	20.163 ±1.095	20.285 ± 1.057	0.122			
SSGs	19.994 ± 1.008	19.150 ± 0.874	−0.844			
Effects	Sum of Squares	df	Mean Square	F	P	η^2^p
Time	2.220	1	2.220	4.020	0.049	0.054
Time x Condition	33.576	2	16.788	16.788	<0.001	0.465
Condition	16.235	2	8.117	6.273	<0.003	0.152
**V**_**max**_ **(km.h** ^**−1**^ **)**						
Descriptive statistics	Pre ± SD	Post ± SD	Mean difference			
SIT	28.582 ± 1.106	29.045 ± 1.216	0.463			
PT	29.35 ± 1.086	28.722 ± 1.141	−0.636			
SSGs	27.889 ± 1.380	27.804 ± 1.215	−0.085			
Effects	Sum of Squares	df	Mean Square	F	P	η^2^p
Time	0.270	1	0.270	0.335	0.565	0.005
Time x Condition	7.386	2	3.693	4.576	0.014	0.016
Condition	38.356	2	19.178	9.365	<0.001	0.211
**MAS (km.h** ^**−1**^ **)**						
Descriptive statistics	Pre ± SD	Post ± SD	Mean difference			
SIT	14.612 ± 1.102	16.354 ± 1.054	1.742			
PT	14.748 ± 1.216	15.330 ± 1.086	0.582			
SSGs	14.358 ± 1.131	13.842 ± 1.091	−0.516			
Effects	Sum of Squares	df	Mean Square	F	P	η^2^p
Time	133.598	1	133.598	133.598	0.013	0.085
Time x Condition	859.325	2	859.325	859.325	<0.001	0.375
Condition	4526.524	2	2263.262	39.732	<0.001	0.532
**D**_**max**_ **(m)**						
Descriptive statistics	Pre ± SD	Post ± SD	Mean difference			
SIT	140.346 ± 7.250	148.231 ± 7.506	7.885			
PT	137.478 ± 5.907	139.174 ± 4.969	1.696			
SSGs	132.750 ± 6.187	128.917 ± 4.966	−3.653			
Effects	Sum of Squares	df	Mean Square	F	P	η^2^p
Time	133.598	1	133.598	6.538	0.013	0.085
Time x Condition	859.325	2	429.663	21.026	<0.001	0.375
Condition	4526.524	2	2263.262	39.732	<0.001	0.532
**D**_**mean**_ **(m)**						
Descriptive statistics	Pre ± SD	Post ± SD	Mean difference			
SIT	136.692 ± 6.981	145.385 ± 4.464	8.693			
PT	118.370 ± 6.941	124.347 ± 5.332	5.977			
SSGs	117.369 ± 10.029	117.506 ± 9.869	0,137			
Effects	Sum of Squares	df	Mean Square	F	P	η^2^p
Time	886.902	1	886.902	67.567	<0.001	0.491
Time x Condition	471.980	2	235.990	17.978	<0.001	0.339
Condition	16100.584	2	8050.292	80.086	<0.001	0.696

*SIT, sprint interval training; TG, plyometric training; SSGs, small-sided games*.

### *V*_0–10 m_

There was a significant loss of speed for the SSG group (−4 ± 5%; *p* < 0.001) when a statistical improvement was found in the SIT group (8 ± 5%; *p* < 0.001). No statistical changes were seen in the PT group (1 ± 6%; *p* = 0.835). A time × condition interaction was observed (*p* < 0.001; *η*^2^*p* = 0.47) for *V*_0−10m_ where the SSG group was significantly lower from PT and SIT groups in POST (*p* < 0.001).

### *V*_max_

Only slight and non-significant changes were obtained in *V*_max_ for SSG and SIT groups. In contrast, *V*_max_ significantly decreased in the PT group (−2 ± 4%; *p* < 0.05). *V*_max_ showed a time × condition interaction (*p* < 0.05; *η*^2^p = 0.12) where the SSG group was significantly different from PT and SIT groups in POST (*p* < 0.05 and *p* < 0.01, respectively).

### *D*_max_

No statistical differences were observed in *P*_max_ for the PT group. There was a significant decrease for the SSG group (−3 ± 6%; *p* < 0.01), while the SIT group showed significant improvement with 6% ± 5% (*p* < 0.001). An interaction time × condition was observed (*p* < 0.001; *η*^2^*p* = 0.38) for P_max_ where SSGs group was significantly different from PT and SIT groups in POST (*p* < 0.001).

### *D*_mean_

There was no significant change for the SSG group (0 ± 5%; *p* = 0.89). In contrast, both PT and SIT groups showed statistical improvements (5 ± 4 and 7 ± 5%, respectively; *p* < 0.001). In *P*_mean_, a time × condition interaction was observed (*p* < 0.001; *η*^2^*p* = 0.34) where the SSG group was significantly lower from PT (*p* < 0.01) and SIT (*p* < 0.001) groups in POST. In addition, PT and SIT groups were also different in POST (*p* < 0.001).

### MAS

A significant increase in MAS was observed for both PT (7 ± 6%; *p* < 0.01) and SIT (12 ± 8%; *p* < 0.001) groups when SSGs registered a significant decrease (−3 ± 6%; *p* < 0.001). An interaction time × condition was observed (*p* < 0.001; *η*^2^*p* = 0.52) in MAS where (i) the SSG group was significantly different from PT and SIT groups in POST (*p* < 0.001) and (ii) PT and SIT groups were also different in POST (*p* < 0.001).

## Discussion

This study demonstrated that PT and SIT improved athletic performance when SSGs did not. More specifically, SIT significantly improved athletic performance (e.g., *V*_0−10m_, *D*_max_, *D*_mean_, and MAS) while PT did not reach such a high impact, and SSGs showed no statistical increases. In fact and contrary to the literature (Impellizzeri et al., 2006; Dellal et al., 2012a; Kunz et al., 2019), the statistical changes in the SSG group were only losses in *V*_0−10m_ (−4%), *P*_max_ (−3%), and MAS (−3%).

### Speed

Our results are in line with Bujalance-Moreno and Garc-a-Pinillos (2017), Jastrzebski et al. (2014), and Hill-Haas et al. (2009) as SIT and SSG training did not affect *V*_max_ but in contrast with Chaouachi et al. (2014) who reported a significant increase in *V*_max_ in both sprints and SSG groups. On shorter distance (i.e., *V*_0−10m_), Chaouachi et al. (2014) also obtained significant improvements after the sprint and SSG training when we found an increase in the SIT group (8%, *p* < 0.001) and a decrease in the SSG group (−4%, *p* < 0.001) where both groups were significantly different in POST. As Bujalance-Moreno et al. (2019) indicated that soccer players could perform up to 250 brief explosive actions during a game, it seems more important to focus on an increase of *V*_0−10m_ rather than *V*_max_ which would be reached very occasionally during the game. Thus, the results obtained in the PT group (loss in *V*_max_ with no changes in *V*_0−10m_) are in accordance with the SSG group in which *V*_max_ did not change but *V*_0−10m_ decreased. Additionally, SIT seems to offer the best compromise with no change in *V*_max_ in POST and a significant increase of *V*_0−10m_.

### RSA

Improvements in sprint performance (see results) and RSA have been identified as determinant factors of team sports (Reilly et al., 2009; Buchheit et al., 2010; Bujalance-Moreno et al., 2019). Rampinini et al. (2009) even suggested that the ability to complete repeated sprints could be one of the best physical factors differentiating the playing level in soccer players. Despite increases that were reported in previous studies after 2–6 weeks of SSG training (Owen et al., 2012; Bujalance-Moreno and Garc-a-Pinillos, 2017; Rodríguez-Fernández et al., 2017), in this study, and as for speed, SSGs had no effect on *D*_mean_ and negatively affected *D*_max_ (−3%). It is important to notice that those results were significantly different from PT and SIT groups where PT presented no change in *D*_max_ (1%) and statistical improvements in *D*_mean_ (5%). Once again, SIT exhibited the highest increase in both *D*_max_ (6%) and *D*_mean_ (7%). Nonetheless, SIT enhancements were in the lower part of the bulk of the literature on 2–4 week SIT which experienced a 5–17% improvement in *D*_max_ and a 4–17% in *D*_mean_ (Burgomaster et al., 2005, 2006; Hazell et al., 2010; Whyte et al., 2010; Bayati et al., 2011; Willoughby et al., 2016). Besides the previously discussed differences in subject training experience, it is important to notice that this study performed tests and training on the field, which do not allow for the same level of accuracy as direct measures of power on a cycle ergometer.

### MAS

Maximal aerobic speed in the SIT group improved significantly by 12% following the 3-week intervention. The PT group also progressed (7%, *p* < 0.01) but slightly less than the SIT group. Compared with the literature that utilized a 2–4-week SIT intervention period (−2 to 9.5% Burgomaster et al., 2006; Iaia et al., 2009; Hazell et al., 2010; Whyte et al., 2010; Bayati et al., 2011; Astorino et al., 2012; Denham et al., 2015; Willoughby et al., 2016), the results of this study are higher. Furthermore, these studies were conducted in untrained or active subjects, and our results are then even more valuable since our participants were already trained soccer players. Potentially, this type of training could have enhanced neuromuscular capacity in soccer players as supported by the improvements obtained in *D*_max_ and *D*_mean_ (see results). It can be assumed that the PT group which also increased both MAS and *D*_mean_ may share part of the adaptive mechanisms. Those may result in a better running economy and therefore higher performances (Helgerud et al., 2001; Rowan et al., 2012). Interestingly, the SSG group presented a significant decrease in MAS (−3%, *p* < 0.001). As players were not wearing any GPS system associated with a heartbeat monitor, the internal training load could not be quantified. Nevertheless, it can be speculated that the technical-tactical level of the players was not good enough to deal with the SSGs proposed by coaches (Dellal et al., 2011, 2012b). Consequently, as proposed by Castagna et al. (2004), players were not able to maintain the required metabolic strain even if the coaches chose SSGs involving possession in which heart rate was supposed to be higher (Castellano et al., 2013). Moreover, contrary to Rampinini et al. (2007b), the 3-min recovery between sets during SSG sessions was passive. This may also have participated to lower much of the metabolic strain. In line with Bujalance-Moreno et al. (2019), another hypothesis is that contrary to SIT and PT, two sessions of SSGs per week over 3 weeks were not enough to develop any change in players or as in our case lead to a significant decrease.

However, players require well-developed aerobic endurance to maintain intense levels of activity and to limit fatigue at the same time (Köklö et al., 2015). Furthermore, improvement in MAS allows for greater involvement with the ball, total distance covered, and an increase in the number of sprints performed during match play (Radziminski et al., 2013). Developing MAS still is one of the main objectives in soccer, and this study highlighted SIT as a very adequate method.

### Limitations

As this study was intentionally field-related, direct measures of VO_2max_ and heart rate were not performed so that the internal load could not be assessed. This is a potential limitation when trying to explain some of the results found notably in the SSG group.

Another point is that even if PT and SIT enhanced the performance in speed, RSA, and MAS, it can only be speculated that participants of PT and SIT groups will be more efficient during a real soccer game. Objectively, it would be hard to determine (i) if those 3–12% improvements depending on the parameter and the training group (PT or SIT) will have a real impact on a game situation, and (ii) what would be the magnitude of real competitive effects due to those improvements.

It could also be speculated that with a longer training time (i.e., 4–6 weeks) and a higher frequency (three times per week), PT would give results that would be closer to SIT.

Moreover, since the SIT group performed training sessions that were close to the RSA testing procedure, the number of repetitions achieved during SIT sessions might have positively influenced the performance in *D*_max_ and *D*_mean_.

Finally, the results reported, in this study, can also be a reflection of faster adaptations in one training compared with the others as it is now well-admitted that adaptations induced by the different training methods require a different amount of time to appear. Future studies should consider this parameter and program different post-testing sessions (i.e., 1 and 2 weeks after the first post-tests).

### Practical Applications

Overall, all performance parameters presented a time × condition interaction where the SSG group was significantly different from PT and SIT groups in POST. More specifically, those interactions expressed significant opposite evolutions where SIT and PT maximized performance parameters where SSGs tended to diminish them. Moreover, they also suggest that SIT seems to be more efficient than PT when attempting to improve *D*_mean_ and MAS.

As a result and contrary to the literature, SSGs, even though they could have some positive aspects on physical performance, should not be considered as a training method but mainly as a way to improve technical-tactical aspects. In this precise case, it is possible that the principle of specificity as defined by Reilly et al. (2009) would not allow reaching maximum benefits. Effectively, if SSGs are very similar to the conditions of a soccer match, they do not always simulate the high-intensity efforts and repeated sprints that the full game demands (Casamichana et al., 2012).

## Conclusion

As modern coaches must constantly deal with an increasingly congested fixture period of competitions which subsequently reduces the preparation time available, the results of this study demonstrate that SIT could be a more efficient alternative than SSGs and PT when aiming to enhance both endurance and anaerobic performances in preseason. In addition, as the ability to complete repeated sprints could be one of the best physical factors differentiating the playing level in soccer players, SIT could be used as an efficient training and as a testing method indifferently.

## Data Availability Statement

The original contributions presented in the study are included in the article/supplementary material, further inquiries can be directed to the corresponding author/s.

## Ethics Statement

The studies involving human participants were reviewed and approved by French Ethics Committee. The patients/participants provided their written informed consent to participate in this study.

## Author Contributions

JK contributed to the conception, design of this study, and drafted the manuscript. FLR collected the data. JK, JLV, FLR, and CF contributed to the analysis and interpretation of the data. JLV, FLR, and CF critically revised the manuscript. All authors gave final approval and agreed to be accountable for all aspects of work ensuring integrity and accuracy.

## Conflict of Interest

The authors declare that the research was conducted in the absence of any commercial or financial relationships that could be construed as a potential conflict of interest.

## Publisher's Note

All claims expressed in this article are solely those of the authors and do not necessarily represent those of their affiliated organizations, or those of the publisher, the editors and the reviewers. Any product that may be evaluated in this article, or claim that may be made by its manufacturer, is not guaranteed or endorsed by the publisher.
